# Determinants of childhood vaccination differ between conflict-affected and non-conflict regions in Cameroon

**DOI:** 10.3389/fpubh.2026.1892579

**Published:** 2026-09-21

**Authors:** Rodolfo Rossi, Siaka Konaté, Amalid Chebly, Benjamin Awouyiegnigni, Kurt Barbé, Ives Hubloue

**Affiliations:** 1 Policy and Research Unit, International Committee of the Red Cross, Geneva, Switzerland; 2 Research Group on Emergency and Disaster Medicine, Vrije Universiteit Brussel, Brussels, Belgium; 3 Health Department, International Committee of the Red Cross, Yaoundé, Cameroon; 4 Health Unit, International Committee of the Red Cross, Geneva, Switzerland; 5 Biostatistics & Medical Informatics Research Group, Vrije Universiteit Brussel, Brussels, Belgium

**Keywords:** Cameroon, conflict-affected settings, Demographic and Health Survey, health inequalities, immunization, vaccination coverage

## Abstract

**Introduction:**

Childhood vaccination remains one of the most effective public health interventions, yet coverage is often lowest in settings affected by conflict and structural disadvantage. Cameroon has faced overlapping humanitarian crises in its Far North, North-West, and South-West regions, but evidence comparing determinants of full vaccination between conflict-affected and non-conflict regions remains limited. This study explores determinants of full vaccination status in 12–23 months children in Cameroon, stratified by conflict regions.

**Methods:**

We conducted a secondary analysis of the 2018 Cameroon Demographic and Health Survey, including 1,824 living children aged 12–23 months. Full basic vaccination was defined as receipt of one dose of BCG, three doses of pentavalent vaccine, three doses of polio vaccine, and one dose of measles vaccine. Survey-weighted logistic regression was used to identify factors associated with full vaccination overall and by conflict status.

**Results:**

Overall, 53.08% of children were fully vaccinated. Coverage was lower in conflict-affected than in non-conflict regions (45.29% versus 55.97%). In unadjusted analysis, conflict status was associated with lower odds of full vaccination, but this association was no longer significant in the adjusted model. At the national level, higher odds of full vaccination were associated with richer household wealth, maternal secondary or higher education, antenatal care attendance, and postnatal care. In conflict-affected regions, maternal secondary or higher education, postnatal care, and facility delivery remained significant predictors of full vaccination.

**Discussion:**

Full vaccination coverage in Cameroon remains below desirable levels, with lower crude coverage in conflict-affected regions. However, after adjustment, conflict status was not independently associated with full vaccination, suggesting that socioeconomic conditions and contact with maternal health services are more important determinants. Strengthening antenatal and postnatal care and reducing socioeconomic barriers may improve vaccination coverage, especially in fragile settings.

## Introduction

Disease prevention through childhood vaccination has been proved to be one of the most effective interventions that saved, and continue to save, million lives worldwide: it is estimated that every year vaccination can avert approximately two million deaths; moreover, vaccination reduces childhood morbidity and disability. Since 1974, when the Expanded Programme of Immunization was launched, it is estimated that vaccination has prevented 146 million deaths among children under 5 years of age (1–3).

Sustained high immunization coverage is essential to maximize the impact of vaccination programs and to protect individuals who cannot be vaccinated. This protection relies on herd immunity, which occurs when a sufficiently large proportion of the population is immune to interrupt pathogen transmission. For highly transmissible diseases such as measles, vaccination coverage of around 95% is generally required to prevent outbreaks. Failure to achieve and maintain such coverage increases the risk of outbreaks, particularly in settings where immunization services are disrupted (4–7).

Maintaining sufficient vaccination coverage to achieve herd immunity thresholds faces multiple challenges. Concurrent pandemics, public health emergencies, population movements, vaccine hesitancy, and misinformation can all disrupt immunization efforts (8–11). Countries affected by armed conflict typically exhibit lower vaccination coverage, higher incidence and mortality from vaccine-preventable diseases, and elevated risk of epidemics (12). These patterns underscore the compounded risks in settings like Cameroon, where ongoing crises in the Far North, North-West, and South-West regions further constrain immunization programmes. In this context, understanding the current level of vaccination coverage is essential. Vaccination coverage among children in Cameroon is still lower than the target set globally: in 2018, national coverage with the first dose of measles-containing vaccine in Cameroon was approximately 61%, well below the ≥95% coverage generally required to prevent measles outbreaks and below the Immunization Agenda 2030 target of at least 90% national coverage (13, 14).

Cameroon is a lower-middle-income country in Central Africa with a population of approximately 28 million people. It is characterized by considerable linguistic, ethnic, and religious diversity, with French and English as its official languages. Despite its natural resources, poverty remains widespread, with approximately 40% of the population living below the national poverty line, and persistent socioeconomic inequalities and ongoing armed conflicts continue to challenge equitable access to healthcare, including childhood immunization (15). Cameroon has experienced overlapping humanitarian crises in its Far North, North-West, and South-West regions over the past decade. In the Far North, armed violence linked to Boko Haram and affiliated groups emerged around 2014, causing recurrent attacks on civilians, population displacement, and a sustained security response by national and regional forces. In the predominantly Anglophone North-West and South-West regions—where approximately 20% of the population resides—protests by lawyers, teachers, and other professional groups began in late 2016 over concerns including language use in courts and schools, perceived marginalization, and central governance structures; these escalated from 2017 into armed confrontation between state security forces and non-state armed groups. These situations have led to substantial internal displacement, restricted access to services, and humanitarian needs across affected areas, with hundreds of thousands of people reported as internally displaced persons and millions requiring assistance (16–19).

Very few studies have been conducted to assess the vaccination coverage and its predictors in Cameroon. Although these studies are methodologically robust and valid, they focused on limited factors, such as the dropout in coverage due to the COVID19 pandemic (20, 21), are limited in terms of geographical areas and sample size (22) or focused on the coverage of a specific antigen (23). However, these studies agree on the fact that childhood vaccination coverage in the Cameroon is low and subjected to decline in case of other emergencies. On the other hand, these studies do not systematically assess various levels of factors associated with childhood vaccination in the country, nor compare regions affected by armed conflict with more stable areas.

The aim of this study is to explore factors associated with childhood vaccination coverage in Cameroon using the most recent countrywide vaccination coverage survey (DHS 2018); to compare such factors between regions affected by armed conflict with regions more stable; to propose strategies to increase vaccination coverage overall and for specific subgroups of populations.

## Materials and methods

### Ethics statement

The Demographic and Health Survey (DHS) of Cameroon conducted between June 2018 and January 2019 (EDSC-V) by the National Institute of Statistics, in close collaboration with the Ministry of Public Health (24). The DHS protocol was reviewed and approved by the ICF Institutional Review Board - ICF IRB FWA00000845 - and by the Cameroon National Ethics Committee for Human Health (CNERSH) -132989.0.000. CM. DHS.01. Written informed consent was obtained from all participants prior to data collection. The datasets used in this study are publicly available and anonymized, and therefore no additional ethical approval was required for this secondary analysis (ICRC Ethics Process exemption reference GVA_CORE_1925). The DHS and ICF granted access to the dataset on the 14 August 2025.

### Materials

The analysis was conducted using data from the 2018 Cameroon Demographic and Health Survey (DHS). The DHS uses a two-stage stratified cluster sampling design to obtain a nationally representative sample of households. In the first stage, clusters (enumeration areas) were selected from the national sampling frame with probability proportional to size. In the second stage, households were randomly selected within each cluster. The survey collected detailed information on maternal and child health, including vaccination history for children born in the five years preceding the survey. This study used the Children’s Recode (KR) dataset. The analysis was restricted to living children, one per household, aged 12–23 months at the time of the survey, which is the standard age group used to assess completion of basic childhood vaccination. The analysis was restricted to children aged 12–23 months because, according to the national immunization schedule, all vaccines included in the definition of full vaccination should have been administered by 12 months of age. Children with missing information on vaccination status were excluded from the corresponding analyses.

### Methods

The outcome variable was full basic vaccination status. A child was considered fully vaccinated if they had received one dose of Bacille Calmette–Guérin (BCG), three doses of pentavalent vaccine, three doses of polio vaccine (excluding the birth dose), and one dose of measles vaccine. Vaccination was considered received if the vaccination date was recorded on the card, the vaccination was marked on the card without a date, or the mother reported that the child had received the vaccine.

The main exposure of interest was residence in a conflict-affected region. The Far North, North-West, and South-West regions were classified as conflict-affected because they have experienced sustained armed conflict during the study period, whereas the remaining seven regions were classified as non-conflict regions. This classification was based on the regional level available in the DHS dataset, which represents the finest geographic resolution accessible for public-use data. All other regions were classified as non-conflict regions. Figure 1 shows the geographical classification of conflict-affected and non-conflict regions of Cameroon. The 2018 DHS was conducted during ongoing armed conflict, and the DHS final report notes that insecurity prevented complete coverage of some areas of the South-West region, resulting in partial under-representation of that region.

**Figure 1:**
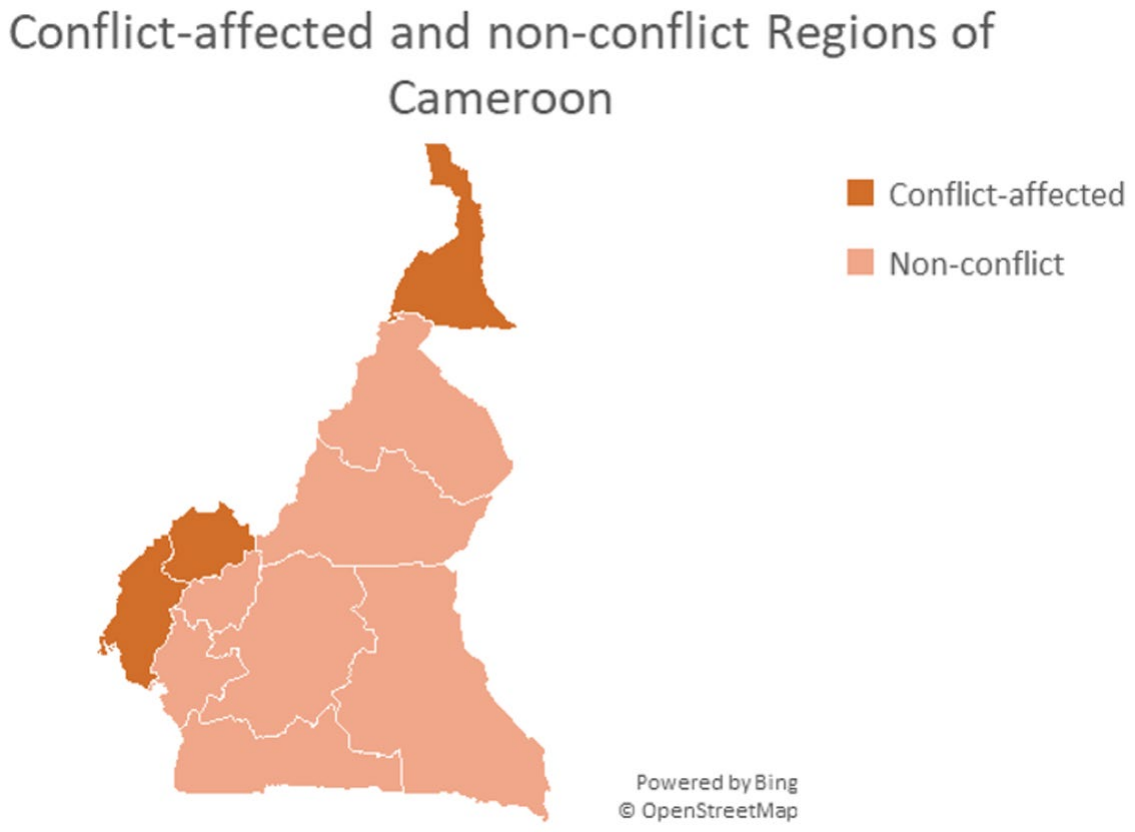
Classification of the ten administrative regions of Cameroon into conflict-affected (Far North, North-West, and South-West) and non-conflict regions used for the analyses.

Potential determinants of full vaccination included child, maternal, household, and health service utilization characteristics, as suggested in previous literature (25). These variables included sex of the child, parity (categorized into two groups using the sample median to provide a simple comparison between women with lower and higher parity and to maintain comparability between groups), maternal age group (15–24, 25–34, and 35–49 years), maternal education (no education, primary, secondary or higher), household wealth quintile (Poorest, Poorer, Middle, Richer or Richest), place of residence (urban or rural), perceived distance to a health facility (big problem vs. not a big problem), number of antenatal care visits during pregnancy (0, 1–3, ≥4 visits), place of delivery (home vs. health facility), and postnatal care for the newborn.

#### Statistical analysis

Descriptive statistics were calculated to summarize the characteristics of the study population. Weighted percentages were reported together with unweighted frequencies. Differences between conflict-affected and non-conflict regions were assessed using design-adjusted Pearson chi-square tests. Associations between explanatory variables and full vaccination were examined using survey-weighted logistic regression models, and statistical significance was evaluated using Wald tests. All analyses accounted for the complex sampling design of the DHS by applying sampling weights and adjusting for clustering at the primary sampling unit level and stratification. Potential interaction effects between selected covariates, including conflict status with maternal education, household wealth, and antenatal care visits, were explored by including multiplicative interaction terms in the survey-weighted logistic regression models. Interaction terms with *p* < 0.05 were considered statistically significant and retained in the final model. As none of the interaction terms reached statistical significance, they were not retained in the final models. Model discrimination was assessed using Tjur’s coefficient of discrimination (Tjur’s R^2^), calculated as the difference between the mean predicted probabilities among fully vaccinated and non-fully vaccinated children. Observations with missing values in variables included in the regression analyses were excluded using complete-case analysis. Missing data were minimal for all study variables, with the exception of antenatal care visits, for which 119 observations (6.5%) had missing information. Bivariate logistic regression analyses were performed to examine the association between each independent variable and full vaccination status. Candidate explanatory variables were selected *a priori* based on previous literature and their potential relevance to childhood vaccination. Among these candidate variables, those associated with the outcome at *p* < 0.20 in bivariable analyses were entered into the multivariable logistic regression models. The *p* < 0.20 threshold was chosen to avoid excluding potentially important confounders during model building (26, 27). Separate multivariable models were estimated for the entire country, for conflict-affected regions, and for non-conflict regions. Multicollinearity among the explanatory variables was assessed using variance inflation factors (VIFs). No evidence of problematic multicollinearity was observed for the three models. Adjusted odds ratios (AORs) and 95% confidence intervals (CIs) were reported. Statistical significance in the final models was defined as a two-sided *p*-value <0.05.

All statistical analyses were performed using Stata (version 16; StataCorp, College Station, TX, USA).

## Results

### Study population and characteristics

A total of 1,824 children aged 12–23 months from the 2018 Cameroon Demographic and Health Survey were included in the analysis (from the initial sample size of 1900 children 76 children were excluded for missing values on the outcome of interest). Of these, 468 children lived in conflict-affected regions (Far North, North-West, and South-West), while 1,356 children lived in non-conflict regions.

The characteristics of the study population overall and by conflict status are presented in Table 1. Overall, 52.12% of children were male, and 56.82% lived in rural areas. Rural residence was considerably more common in conflict-affected regions (74.55%) than in non-conflict regions (50.17%). Household wealth distribution also differed significantly between regions (*p* < 0.001), with children in conflict-affected areas more frequently belonging to the poorest wealth quintile (36.70%) compared with those in non-conflict regions (15.42%). Vaccination cards were seen by interviewers for 71.1% of children overall. Card availability differed significantly by conflict status, with cards being seen for 59.7% of children in conflict-affected regions compared with 73.6% in non-conflict regions (*p* = 0.004).

**Table 1:** Characteristics of study participants for the whole country, conflict-affected and non-conflict regions.

Characteristics	Overall *N* = 1824	Conflict-affected *N* = 468	Non-conflict *N* = 1,356
Sex of child	*p* = 0.210		
Female	883 (47.88%)	228 (45.13%)	655 (51.09%)
Male	941 (52.12%)	240 (54.87%)	701 (48.91%)
Residence	*p* < 0.001		
Rural	1,032 (56.82%)	322 (74.55%)	710 (50.17%)
Urban	792 (43.18%)	146 (25.45%)	646 (49.83%)
Wealth index	*p* < 0.001		
Poorest	321 (21.22%)	157 (36.70%)	164 (15.42%)
Poorer	444 (24.30%)	118 (25.38%)	326 (23.90%)
Middle	453 (21.47%)	101 (20.81%)	352 (21.72%)
Richer	341 (17.55%)	58 (10.67%)	283 (20.12%)
Richest	265 (15.45%)	34 (6.45%)	231 (18.83%)
Mother’s education	*p* < 0.001		
No education	401 (26.00%)	213 (47.20%)	188 (18.06%)
Primary	599 (31.27%)	147 (29.26%)	452 (32.02%)
Secondary or higher	824 (42.73%)	108 (23.54%)	716 (49.92%)
Mother’s age	*p* = 0.488		
15–24	699 (37.09%)	166 (34.46%)	533 (38.07%)
25–34	841 (46.83%)	223 (48.46%)	618 (46.22%)
35+	284 (16.08%)	79 (17.08%)	205 (15.71%)
Parity	*p* = 0.447		
Low parity (1–3)	1,094 (58.63%)	261 (56.91%)	833 (59.27%)
High parity (4+)	730 (41.37%)	207 (43.09%)	523 (40.73%)
Antenatal care visits (*N* = 1705)	*p* = 0.008		
0 ANC visits	208 (12.53%)	90 (18.17%)	118 (10.37%)
1–3 ANC visits	384 (21.32%)	89 (20.08%)	295 (21.79%)
≥4 ANC visits	1,113 (66.15%)	264 (61.75%)	849 (67.83%)
Place of delivery	*p* < 0.001		
Health facility	1,298 (68.63%)	265 (54.95%)	1,033 (73.76%)
Home	526 (31.37%)	203 (45.05%)	323 (26.24%)
Postnatal care for newborns	*p* = 0.772		
Yes	493 (30.40%)	125 (31.24%)	368 (30.08%)
No	1,218 (69.60%)	316 (68.76%)	902 (69.92%)
Distance to health facility	*p* = 0.055		
Not a big problem	1,060 (58.49%)	244 (53.13%)	816 (60.50%)
Big problem	764 (41.51%)	224 (46.87%)	540 (39.50%)

Frequencies are unweighted, percentages are weighted for survey design. *p*-values from Pearson Chi2 Test for the difference between conflict-affected versus non-conflict regions. Data are presented as *n* (weighted %). Frequencies are unweighted and percentages account for the survey design.

### Vaccination coverage

Vaccination coverage among children aged 12–23 months is shown in Table 2. Overall coverage was 86.74% for BCG, 85.17% for polio dose 1, 68.00% for polio dose 3, 82.86% for pentavalent dose 1, 72.84% for pentavalent dose 3, and 65.90% for measles vaccination.

**Table 2:** Vaccination coverage of children aged 12–23 months in the whole country, in conflict-affected and non-conflict regions.

Vaccine	Overall *N* = 1824, *n* (%)	Conflict-affected *N* = 468, *n* (%)	Non-conflict *N* = 1,356, *n* (%)	*p*-value*
BCG	1,583 (86.74)	377 (81.18)	1,206 (88.81)	0.0203
Polio 1	1,545 (85.17)	375 (81.17)	1,170 (86.66)	0.0229
Polio 3	1,247 (68.00)	273 (60.63)	974 (70.75)	0.0091
Penta 1	1,503 (82.86)	363 (78.22)	1,140 (84.60)	0.0445
Penta 3	1,328 (72.84)	301 (65.40)	1,027 (75.62)	0.0114
Measles	1,198 (65.90)	277 (59.61)	921 (68.25)	0.0259
Fully vaccinated§	977 (53.08)	200 (45.29)	777 (55.97)	0.0211

Frequencies are unweighted, percentages are weighted for survey design. ^*^ *p*-values from Pearson Chi2 Test for the difference between conflict-affected versus non-conflict regions. §Fully vaccinated: received 1 dose BCG, 3 Pentavalent, 3 Polio, 1 Measles.

Overall, 53.08% of children were fully vaccinated according to the basic vaccination schedule.

Coverage was consistently lower in conflict-affected regions compared with non-conflict regions. In particular, full vaccination coverage was 45.29% in conflict-affected regions compared with 55.97% in non-conflict regions (*p* = 0.021). Similarly, coverage for polio dose 3 (60.63% vs. 70.75%) and pentavalent dose 3 (65.40% vs. 75.62%) was significantly lower in conflict regions.

### Determinants of full vaccination in the whole country

In bivariate analyses, several socioeconomic and healthcare utilization factors were associated with full vaccination, including residence, household wealth, maternal education, parity, antenatal care attendance, postnatal care for the newborn, place of delivery, and perceived distance to a health facility. In the multivariable model, household wealth, maternal education, antenatal care visits, and postnatal care for the newborn remained significantly associated with full vaccination. Children from the richest households were significantly more likely to be fully vaccinated compared with those from the poorest households (aOR = 1.81; 95% CI: 1.01–3.24; *p* = 0.046). Similarly, children whose mothers had secondary or higher education were more likely to be fully vaccinated than those whose mothers had no education (aOR = 1.64; 95% CI: 1.13–2.40; *p* = 0.010).

Utilization of maternal health services was also strongly associated with vaccination completion. Compared with children whose mothers had no antenatal care visits, those whose mothers attended 1–3 antenatal visits (aOR = 2.54; 95% CI: 1.56–4.14; *p* < 0.001) or four or more visits (aOR = 2.68; 95% CI: 1.64–4.38; *p* < 0.001) had significantly higher odds of being fully vaccinated. In addition, children who received postnatal care were more likely to be fully vaccinated (aOR = 1.64; 95% CI: 1.24–2.18; *p* = 0.001). Although full vaccination coverage was lower among children living in conflict-affected regions in the descriptive analyses, conflict status was not independently associated with full vaccination after adjustment for socioeconomic, maternal, and healthcare-related factors. Results of the logistic regression analyses for the entire country are presented in Table 3.

**Table 3:** Bi- and multivariable logistic regression for factors associated with full basic childhood vaccination for the whole country.

Predictor (category)	Bivariate logistic regression		Multivariable logistic regression	
	cOR (95% CI)	*p*-value	aOR (95%CI)	*p*-value
Conflict status				
Conflict-affected	0.65 (0.45–0.94)	0.022	0.91 (0.64–1.29)	0.579
Non-conflict	Ref	--	Ref	--
Sex of child				
Male	Ref	--		
Female	0.97 (0.77–1.21)	0.790		
Residence				
Urban	Ref	--	Ref	--
Rural	0.54 (0.41–0.71)	<0.001	0.93 (0.65–1.33)	0.686
Wealth quintile				
Poorest	Ref	--	Ref	--
Poorer	1.80 (1.24–2.62)	0.002	1.26 (0.83–1.92)	0.279
Middle	2.09 (1.45–3.00)	<0.001	1.25 (0.83–1.89)	0.281
Richer	2.89 (1.96–4.25)	<0.001	1.48 (0.87–2.52)	0.143
Richest	4.54 (2.91–7.09)	<0.001	1.81 (1.01–3.24)	0.046
Mother’s education				
No education	Ref	--	Ref	--
Primary	1.44 (1.02–2.03)	0.039	0.94 (0.66–1.34)	0.751
Secondary or higher	3.47 (2.48–4.87)	<0.001	1.64 (1.13–2.40)	0.010
Mother’s age				
15–24	Ref	--		
25–34	1.13 (0.90–1.42)	0.300		
35+	1.03 (0.74–1.43)	0.870		
Parity*				
High parity	Ref	--	Ref	--
Low parity	0.77 (0.62–0.95)	0.014	1.08 (0.86–1.35)	0.504
ANC visits				
0 ANC visits	Ref	--	Ref	--
1–3 ANC visits	3.48 (2.22–5.45)	<0.001	2.54 (1.56–4.14)	<0.001
≥4 ANC visits	4.96 (3.21–7.64)	<0.001	2.68 (1.64–4.38)	<0.001
PNC				
No	Ref	--	Ref	--
Yes	1.95 (1.50–2.52)	<0.001	1.64 (1.24–2.18)	0.001
Place of delivery				
Home	Ref	--	Ref	--
Health facility	2.72 (2.05–3.60)	<0.001	1.29 (0.92–1.81)	0.146
Distance to facility				
Big problem	Ref	--	Ref	--
Not a big problem	1.42 (1.14–1.77)	0.002	0.97 (0.75–1.25)	0.808

Outcome: full basic vaccination (BCG + 3 × Penta + 3 × Polio + Measles). Reported are odds ratios (OR) with 95% confidence intervals and Wald test *p*-values. Tjur’s R^2^ = 0.101. Models are weighted using DHS sampling weights (v005/1,000,000) with cluster-robust standard errors at the PSU level (v001). *Parity defined as number of children ever born; categorized at the sample median (≤3 vs ≥4 children). PNC, Postnatal care for babies (any consultation).

### Determinants of full vaccination in conflict-affected regions

The results of the regression analyses restricted to conflict-affected regions are presented in Table 4.

**Table 4:** Bi- and multivariable logistic regression for factors associated with full basic childhood vaccination for the conflict-affected regions.

Predictor (category)	Bivariate logistic regression		Multivariable logistic regression	
	cOR (95% CI)	*p*-value	aOR (95%CI)	*p*-value
Sex of child				
Male	Ref	--		
Female	0.76 (0.48–1.20)	0.235		
Residence				
Urban	Ref	--		
Rural	0.74 (0.42–1.33)	0.312		
Wealth quintile				
Poorest	Ref	--	Ref	--
Poorer	1.55 (0.82–2.96)	0.178	1.42 (0.71–2.82)	0.317
Middle	1.24 (0.73–2.11)	0.426	0.86 (0.47–1.58)	0.627
Richer	1.99 (0.98–4.08)	0.058	1.09 (0.43–2.75)	0.856
Richest	3.72 (1.55–8.96)	0.004	0.85 (0.30–2.44)	0.766
Mother’s education				
No education	Ref	--	Ref	--
Primary	1.32 (0.67–2.59)	0.417	0.89 (0.48–1.62)	0.691
Secondary or higher	5.93 (3.04–11.60)	<0.001	3.46 (1.62–7.41)	0.002
Mother’s age				
15–24	Ref	--		
25–34	1.02 (0.61–1.69)	0.948		
35+	1.00 (0.52–1.89)	0.990		
Parity*				
High parity	Ref	--	Ref	--
Low parity	0.62 (0.41–0.91)	0.017	0.93 (0.61–1.42)	0.724
ANC visits				
0 ANC visits	Ref	--	Ref	--
1–3 ANC visits	2.25 (1.03–4.88)	0.041	1.45 (0.64–3.32)	0.371
≥4 ANC visits	4.07 (1.76–9.44)	0.001	1.91 (0.81–4.51)	0.136
PNC				
No	Ref	--	Ref	--
Yes	2.06 (1.24–3.42)	0.006	1.82 (1.04–3.20)	0.036
Place of delivery				
Home	Ref	--	Ref	--
Health facility	3.12 (1.82–5.36)	<0.001	1.80 (1.01–3.22)	0.047
Distance to facility				
Big problem	Ref	--	Ref	--
Not a big problem	1.55 (0.98–2.47)	0.062	0.89 (0.53–1.49)	0.657

Outcome: full basic vaccination (BCG + 3 × Penta + 3 × Polio + Measles). Reported are odds ratios (OR) with 95% confidence intervals and Wald test *p*-values. Tjur’s R^2^ = 0.161. Models are weighted using DHS sampling weights (v005/1,000,000) with cluster-robust standard errors at the PSU level (v001). *Parity defined as number of children ever born; categorized at the sample median (≤3 vs ≥4 children). PNC, Postnatal care for babies (any consultation).

In the multivariable model, maternal education, postnatal care for the newborn, and place of delivery remained significantly associated with full vaccination. Children whose mothers had secondary or higher education were significantly more likely to be fully vaccinated compared with those whose mothers had no education (aOR = 3.46; 95% CI: 1.62–7.41; *p* = 0.002).

Similarly, children who received postnatal care had higher odds of being fully vaccinated (aOR = 1.82; 95% CI: 1.04–3.20; *p* = 0.036). Birth in a health facility was also associated with higher odds of full vaccination (aOR = 1.80; 95% CI: 1.01–3.22; *p* = 0.047).

### Determinants of full vaccination in non-conflict regions

Results for non-conflict regions are presented in Table 5. In the adjusted model, household wealth, antenatal care attendance, and postnatal care for the newborn remained significantly associated with full vaccination. Children from the richest households were more than twice as likely to be fully vaccinated compared with those from the poorest households (aOR = 2.44; 95% CI: 1.16–5.10; *p* = 0.018). Utilization of maternal health services was also strongly associated with vaccination completion. Compared with children whose mothers had no antenatal care visits, those whose mothers attended 1–3 visits (aOR = 3.44; 95% CI: 1.86–6.33; *p* < 0.001) or four or more visits (aOR = 3.21; 95% CI: 1.79–5.76; *p* < 0.001) had significantly higher odds of full vaccination. In addition, children who received postnatal care were more likely to be fully vaccinated (aOR = 1.67; 95% CI: 1.20–2.33; *p* = 0.003).

**Table 5:** Bi- and multivariable logistic regression for factors associated with full basic childhood vaccination for the non-conflict regions.

Predictor (category)	Bivariate logistic regression		Multivariable logistic regression	
	cOR (95% CI)	*p*-value	aOR (95%CI)	*p*-value
Sex of child				
Male	Ref	--		
Female	1.04 (0.80–1.36)	0.743		
Residence				
Urban	Ref	--	Ref	--
Rural	0.53 (0.39–0.71)	<0.001	0.85 (0.56–1.29)	0.444
Wealth quintile				
Poorest	Ref	--	Ref	--
Poorer	1.99 (1.22–3.25)	0.006	1.36 (0.76–2.44)	0.295
Middle	2.64 (1.64–4.26)	<0.001	1.67 (0.93–3.00)	0.088
Richer	3.30 (2.07–5.28)	<0.001	1.91 (0.97–3.80)	0.063
Richest	4.98 (2.94–8.42)	<0.001	2.44 (1.16–5.10)	0.018
Mother’s education				
No education	Ref	--	--	
Primary	1.34 (0.91–1.97)	0.136	0.84 (0.56–1.26)	0.389
Secondary or higher	2.80 (1.92–4.08)	<0.001	1.25 (0.82–1.91)	0.305
Mother’s age				
15–24	Ref	--		
25–34	1.19 (0.92–1.54)	0.178		
35+	1.06 (0.71–1.58)	0.770		
Parity*				
High parity	Ref	--	Ref	--
Low parity	0.85 (0.66–1.09)	0.188	1.20 (0.93–1.56)	0.167
ANC visits				
0 ANC visits	Ref	--	Ref	--
1–3 ANC visits	4.02 (2.31–6.99)	<0.001	3.44 (1.86–6.33)	<0.001
≥4 ANC visits	5.27 (3.20–8.67)	<0.001	3.21 (1.79–5.76)	<0.001
PNC				
No	Ref	--	Ref	--
Yes	1.94 (1.43–2.63)	<0.001	1.67 (1.20–2.33)	0.003
Place of delivery				
Home	Ref	--	Ref	--
Health facility	2.40 (1.72–3.35)	<0.001	1.06 (0.68–1.66)	0.785
Distance to facility				
Big problem	Ref	--	Ref	--
Not a big problem	1.33 (1.04–1.72)	0.026	0.97 (0.72–1.30)	0.840

Outcome: full basic vaccination (BCG + 3 × Penta + 3 × Polio + Measles). Reported are odds ratios (OR) with 95% confidence intervals and Wald test *p*-values. Tjur’s R^2^ = 0.104. Models are weighted using DHS sampling weights (v005/1,000,000) with cluster-robust standard errors at the PSU level (v001). *Parity defined as number of children ever born; categorized at the sample median (≤3 vs ≥4 children). PNC, Postnatal care for babies (any consultation).

### Interaction analysis

Potential interaction effects between selected determinants were tested, including interactions between antenatal care attendance and place of delivery, residence and distance to a health facility, and conflict status with maternal education, household wealth, and antenatal care visits. None of the interaction terms reached statistical significance, and interaction terms were therefore not included in the final models. Model discrimination was modest overall, with Tjur’s R^2^ values ranging from 0.10 to 0.16 across the final multivariable models.

## Discussion

### Summary of main findings

This study examined the determinants of full basic childhood vaccination among children aged 12–23 months in Cameroon using nationally representative data from the 2018 Demographic and Health Survey. Overall, just over half of children were fully vaccinated, and vaccination coverage was consistently lower in conflict-affected regions compared with non-conflict regions. In the national model, full vaccination was significantly associated with household wealth, maternal education, antenatal care attendance, and postnatal care for the newborn. An important finding of this study is that, although children living in conflict-affected regions had lower crude vaccination coverage than those living in non-conflict regions, conflict status itself was not independently associated with full vaccination after adjustment. This suggests that the observed disparity in vaccination coverage may be largely explained by differences in socioeconomic conditions and access to maternal and child health services rather than conflict exposure alone. These findings highlight the importance of addressing the underlying structural barriers to immunization in conflict-affected settings, while recognizing that conflict may still indirectly influence vaccination through its effects on healthcare delivery and household vulnerability.

### Differences between conflict and non-conflict regions

This study also provides important insights into how determinants of vaccination differ between conflict-affected and non-conflict regions. Children living in conflict-affected regions had lower vaccination coverage overall, which is consistent with previous evidence showing that armed conflict can disrupt health systems, reduce access to services, and undermine routine immunization programs (9, 12). In addition, attacks against healthcare workers, vaccinators and health facilities, as well as insecurity limiting the movement of healthcare personnel, may further disrupt routine immunization activities and reduce access to vaccination services in conflict-affected settings (28, 29). Our findings are also consistent with reports from Cameroon documenting reductions in routine immunization coverage during periods of insecurity, particularly in the South-West region, where armed conflict has challenged the delivery of vaccination services and supplementary immunization activities (20–22, 30). Conflict status was no longer independently associated with full vaccination after adjustment for socioeconomic and healthcare-related factors. However, this finding should not be interpreted as evidence that conflict has no influence on childhood immunization. Rather, it suggests that the crude association was largely explained by differences in socioeconomic characteristics and access to maternal healthcare services, which may themselves represent pathways through which conflict influences childhood vaccination. This interpretation is consistent with the broader literature showing that armed conflict affects immunization primarily through disruption of health services, displacement, reduced access to care, and deterioration of socioeconomic conditions (9, 12). One possible explanation is that the extensive humanitarian response implemented in conflict-affected regions, in collaboration with local health authorities, may have mitigated some of the adverse effects of conflict on routine immunization services (30–32). However, this hypothesis could not be directly evaluated using the available DHS data and should therefore be interpreted with caution.

In conflict settings, maternal education and healthcare contact points such as postnatal care and facility delivery appeared particularly important for vaccination uptake. These factors may serve as critical entry points for maintaining vaccination coverage in contexts where health systems are weakened and access to routine services is limited. In contrast, in non-conflict regions socioeconomic inequalities and maternal health service utilization remained the dominant determinants of vaccination. This suggests that while the structural determinants of vaccination remain broadly similar across settings, the relative importance of specific factors may differ depending on the context. This pattern is also consistent with observations from other conflict-affected African settings, including Northeast Nigeria, where caregivers identified disruption of health services, insecurity, and continued engagement with healthcare providers as key factors influencing childhood vaccination uptake (11, 12).

### Socioeconomic inequalities in vaccination coverage

Our findings highlight the important role of socioeconomic factors in determining childhood vaccination coverage. Children from wealthier households had significantly higher odds of being fully vaccinated compared with those from poorer households, particularly in non-conflict regions. These findings are also in agreement with a recent DHS-based analysis from Cameroon, which identified maternal education and healthcare utilization as major determinants of childhood vaccination, although that study focused on pentavalent vaccine uptake rather than complete basic vaccination and did not specifically examine differences between conflict-affected and non-conflict regions (23). Similar socioeconomic gradients in vaccination coverage have been documented in many low- and middle-income countries, where economic resources influence access to health services, transportation, and information about preventive care (33, 34).

Maternal education also emerged as an important determinant of vaccination. Children whose mothers had secondary or higher education were significantly more likely to be fully vaccinated, especially in conflict-affected regions. Educated mothers may have greater awareness of the benefits of immunization, better understanding of vaccination schedules, and increased ability to navigate health systems. Previous studies using DHS data from sub-Saharan Africa have consistently reported positive associations between maternal education and childhood vaccination uptake (33–35).

### Importance of health service utilization

Utilization of maternal health services was strongly associated with vaccination completion. Antenatal care attendance was one of the strongest predictors of full vaccination in both the national and non-conflict models. Mothers who attended antenatal care visits were substantially more likely to have children who completed the basic vaccination schedule. Antenatal care provides important opportunities for health education and counseling, including information about the importance of childhood immunization and the recommended vaccination schedule. Antenatal care attendance has also been proved to increase the likelihood of other mother–child health outcomes, such as delivery in health facility and start breastfeeding of the baby (36, 37).

Postnatal care for the newborn was also significantly associated with vaccination completion in all models. Postnatal visits represent critical contact points between families and the health system, during which healthcare providers can reinforce immunization messages, verify vaccination status, and facilitate timely follow-up vaccinations. The lower availability of vaccination cards observed in conflict-affected regions may also reflect broader disruptions in continuity of healthcare. Insecurity, population displacement, and interruptions in routine health services may compromise the retention of personal health records, making it more difficult for caregivers and healthcare providers to verify previous vaccinations and ensure timely completion of the recommended immunization schedule.

In conflict-affected regions, facility-based delivery was an additional determinant of full vaccination. Health facility delivery may facilitate early contact with vaccination services, including administration of birth-dose vaccines and registration into routine immunization programs. An important consideration is that some healthcare utilization variables included in the adjusted models, including antenatal care, postnatal care, and place of delivery, may lie on the causal pathway between conflict exposure and childhood vaccination. Consequently, adjustment for these variables may have attenuated the association between conflict status and full vaccination. However, because the objective of this study was to identify determinants of full vaccination rather than estimate the total causal effect of conflict, these variables were retained in the multivariable analyses. Future studies using causal inference methods may help disentangle the direct and indirect effects of conflict on childhood vaccination.

Numerous studies conducted in Africa and other Low-Income countries showed similar associations between service utilization and childhood vaccination status, in line with the findings of this present study (33, 37, 38).

### Strengths and limitations

This study has some strengths. To our knowledge, this is the first study conducted in Cameroon that assess predictors of childhood vaccination by comparing regions affected by armed conflict against non-affected regions. It uses nationally representative DHS data, allowing the findings to be generalized to the population of Cameroon. The analysis also accounted for the complex sampling design of the DHS and examined determinants of vaccination separately in conflict-affected and non-conflict regions, providing important contextual insights.

However, several limitations should be considered. First, the cross-sectional nature of DHS data limits the ability to infer causal relationships. Second, vaccination status partly relied on maternal recall when vaccination cards were unavailable, which may introduce recall bias. Vaccination cards were less frequently available in conflict-affected than in non-conflict regions, potentially increasing the risk of differential misclassification between the two settings; however, the direction and magnitude of any resulting bias cannot be determined. Third, the analysis was restricted to children aged 12–23 months and may not capture vaccination dynamics among younger children. Fourth, conflict exposure was defined at the regional level because the public DHS dataset does not provide more granular geographic identifiers. Consequently, the analysis could not account for heterogeneity in conflict intensity or duration within the conflict-affected regions, and some degree of exposure misclassification is possible. In addition, the 2018 DHS fieldwork was conducted during ongoing armed conflict. Although the DHS report specifically documents incomplete coverage of some areas of the South-West region because of insecurity, accessibility challenges may also have affected the representativeness of populations living in other conflict-affected areas. Consequently, households residing in the most insecure settings may have been underrepresented, potentially introducing accessibility and selection bias and leading to an underestimation of the impact of conflict on childhood vaccination. Moreover, information on health system factors such as vaccine supply, cold chain equipment, potential role of community health workers and service availability was not available in the dataset. The difference in sample size, with a much smaller sample of children in conflict-affected regions, may have hidden real predictors of full vaccination. Complete-case analysis was used for the multivariable models. Although this approach may introduce bias if data are not missing completely at random, only antenatal care visits had missing data (6.5%), making substantial bias unlikely. Finally, despite the number of variables included in the analyses, residual confounding cannot be ruled out.

### Public health implications

Achieving high and equitable vaccination coverage remains a central priority of global immunization strategies, including the Immunization Agenda 2030 and the Gavi zero-dose agenda, which aim to reach children who have not received even a single routine vaccine dose. Despite substantial progress in expanding immunization programs worldwide, many children in low- and middle-income countries remain incompletely vaccinated, particularly those living in disadvantaged or fragile settings (39).

The findings of this study have important implications for immunization policy and practice in Cameroon. Although conflict status was not independently associated with full vaccination after adjustment, vaccination coverage remained substantially lower in conflict-affected regions. This highlights the need to strengthen immunization services in these settings while simultaneously addressing the socioeconomic and healthcare-related barriers that contribute to lower vaccination uptake. In Cameroon, insecurity, population displacement, reduced functionality of health facilities, transportation constraints and difficulties conducting outreach activities have all affected the delivery of routine immunization services, particularly in the Northwest and Southwest regions (40).

Strengthening maternal health services, particularly antenatal care, postnatal care and facility-based delivery, may provide important opportunities to improve childhood immunization coverage. Integrating vaccination counseling and vaccination status assessment into maternal health services could increase caregivers’ awareness of the vaccination schedule, facilitate early identification of under-immunized children and improve completion of routine vaccination.

The findings also support targeted strategies to reduce socioeconomic barriers to vaccination. Outreach services, mobile vaccination teams and community health workers remain particularly important for reaching children from poorer households and those living in rural, underserved or conflict-affected communities. Ensuring the safety and protection of healthcare workers and vaccination teams is equally important, as attacks against health personnel and insecurity may substantially disrupt immunization activities and limit access to essential health services in conflict settings (28, 29). These findings support ongoing efforts in Cameroon, including implementation of The Big Catch-Up, which prioritizes the identification and vaccination of zero-dose and under-immunized children, particularly in conflict-affected and hard-to-reach districts (41, 42). Vaccination campaigns may also increase coverage in specific circumstances but should complement, rather than replace, routine immunization services (10). Community health workers can be an important resource, mostly in the role of promotion and dissemination of vaccination activities.

Although these recommendations are directly relevant to Cameroon, they are also applicable to other conflict-affected settings where insecurity compounds existing health system weaknesses and socioeconomic disadvantage. Sustaining these interventions will require continued investment in routine immunization systems, community outreach, health workers and primary healthcare services. Recent reductions in international health and humanitarian financing may disproportionately affect fragile and conflict-affected settings, potentially threatening progress toward reducing the number of zero-dose and under-immunized children (43).

## Conclusion

In conclusion, full basic childhood vaccination coverage in Cameroon remains suboptimal and is lower in conflict-affected regions. Socioeconomic factors and utilization of maternal health services are key determinants of vaccination completion. Strengthening antenatal and postnatal care services and addressing socioeconomic barriers may improve vaccination coverage, particularly in conflict-affected areas.

## Data Availability

Publicly available datasets were analyzed in this study. This data can be found at: https://dhsprogram.com/data/available-datasets.cfm, Demographic and Health Surveys program.

## References

[1] Shattock AJ Johnson HC Sim SY Carter A Lambach P Hutubessy RCW . Contribution of vaccination to improved survival and health: modelling 50 years of the expanded Programme on immunization. Lancet. (2024) 403:2307–16. doi: 10.1016/S0140-6736(24)00850-X PMC11140691 38705159

[2] PLOS One. (2026) Ranking 93 health interventions for low- and middle-income countries by cost-effectiveness. Available online at: https://journals.plos.org/plosone/article?id=10.1371/journal.pone.0182951) (Accessed June 27, 2026) 10.1371/journal.pone.0182951 PMC5552255 28797115

[3] World Health Organization. (2026). Global vaccine coverage and childhood survival estimates: 1990–2019. Available online at: https://iris.who.int/items/28f41a72-e09d-433b-9a4d-fbb497d4a16d (Accessed June 27, 2026) 10.2471/BLT.23.290129 PMC10976869 38562199

[4] Oxford Academic (2026). “Herd Immunity”: A Rough Guide | Clinical Infectious Diseases. Available online at: https://academic.oup.com/cid/article-abstract/52/7/911/299077?redirectedFrom=fulltext (Accessed June 27, 2026)

[5] Guerra FM Bolotin S Lim G Heffernan J Deeks SL Li Y . The basic reproduction number (R0) of measles: a systematic review. Lancet Infect Dis. (2017) 17:e420–8. doi: 10.1016/S1473-3099(17)30307-9 28757186

[6] Plans-Rubió P. (2026). Evaluation of the Establishment of herd Immunity in the Population by means of Serological Surveys and Vaccination Coverage. Available online at: https://www.tandfonline.com/doi/full/10.4161/hv.18444 (Accessed June 27, 2026) 10.4161/hv.18444 22426372

[7] Truelove SA Graham M Moss WJ Metcalf CJE Ferrari MJ Lessler J. Characterizing the impact of spatial clustering of susceptibility for measles elimination. Vaccine. (2019) 37:732–41. doi: 10.1016/j.vaccine.2018.12.012, 30579756 PMC6348711

[8] Dalton M Sanderson B Robinson LJ Homer CSE Pomat W Danchin M . Impact of COVID-19 on routine childhood immunisations in low- and middle-income countries: a scoping review. PLoS Glob Public Health. (2023) 3:e0002268. doi: 10.1371/journal.pgph.0002268 PMC10446229 37611014

[9] Chepkurui V Amponsah-Dacosta E Haddison EC Kagina BM. Characterization of national immunization programs in the context of public health emergencies: a case study of 13 countries in the WHO Africa region. Front Public Health. (2021) 9:736532. doi: 10.3389/fpubh.2021.736532 PMC8505981 34650952

[10] Rossi R Assaad R Rebeschini A Hamadeh R. Vaccination coverage cluster surveys in middle Dreib – Akkar, Lebanon: comparison of vaccination coverage in children aged 12-59 months pre- and post-vaccination campaign. PLoS One. (2016) 11:e0168145. doi: 10.1371/journal.pone.0168145 PMC5167265 27992470

[11] Abreu L Schrage P Adeyanju GC Jalo RI Abulfathi AA Bello MM . Caregivers´ qualitative insights on trust, resilience and vaccination attitudes shaping child health in conflict-affected Northeast Nigeria. Confl Heal. (2026) 20:38. doi: 10.1186/s13031-026-00753-w, 41764511 PMC13049947

[12] Grundy J Biggs B-A. The impact of conflict on immunisation coverage in 16 countries. Int J Health Policy Manag. (2019) 8:211–21. doi: 10.15171/ijhpm.2018.127 PMC6499911 31050966

[13] WHO. (2026). WHO Immunization Data portal - African region. Immunization data. Available online at: https://immunizationdata.who.int/dashboard/regions/african-region (Accessed July 23, 2026)

[14] WHO. (2026) WHO Immunization Data portal - Detail page. Immunization data. Available online at: https://immunizationdata.who.int/global/wiise-detail-page (Accessed July 23, 2026)

[15] World Bank. (2026) Overview. Available online at: https://www.worldbank.org/en/country/cameroon/overview (Accessed July 23, 2026)

[16] OCHA. (2023). Cameroon: humanitarian dashboard (January to march 2023) - Cameroon | ReliefWeb. Available online at: https://reliefweb.int/report/cameroon/cameroon-humanitarian-dashboard-january-march-2023 (Accessed June 27, 2026)

[17] ReliefWeb. (2026). UNFPA Cameroon situation report: politics, conflict, climate disaster, and displacement (1 - 31 January 2026) - Cameroon. Available online at: https://reliefweb.int/report/cameroon/unfpa-cameroon-situation-report-politics-conflict-climate-disaster-and-displacement-1-31-january-2026 (Accessed June 27, 2026)

[18] Ngwang A. Wong K. L. M. Banke-Thomas A. (2026). Geospatial analysis of the impact of Cameroonian Anglophone crisis conflict intensity on antenatal care utilisation | Commun med. Available online at: https://www.nature.com/articles/s43856-026-01374-3 (Accessed June 27, 2026) 10.1038/s43856-026-01374-3 PMC12901020 41530305

[19] Susan EB Matilda AB Epey NE. The Anglophone crisis in Cameroon and sustainable development: a causal-process tracing approach. Sustain Dev. 32:6903–17. doi: 10.1002/sd.3058

[20] Saidu Y Di Mattei P Nchinjoh SC Edwige NN Nsah B Muteh NJ . The hidden impact of the COVID-19 pandemic on routine childhood immunization coverage in Cameroon. Vaccines. (2023) 11:645. doi: 10.3390/vaccines11030645 PMC10058217 36992229

[21] Binyet MAO Tchopba CN Beavogui FT Tawambe PB Quashie PK Debnet J . The impact of COVID-19 stringency policies on primary health care in Cameroon: a case study of the expanded program of immunisation. J Glob Health Econ Policy. (2024) 4:e2024016. doi: 10.52872/001c.126658

[22] Ewang BF Eyong ME Cumber SN Nkfusai CN Yankam BM Anye CS . Vaccination coverage under the expanded program on immunization in south West Cameroon. Int J Matern Child Health AIDS. (2020) 9:242–51. doi: 10.21106/ijma.308 PMC7359757 32685280

[23] Bashombwa AM Tchio-Nighie KH Nanfak A Nkum CB Nguemnang WAN Kami RF . Determinants of Access to the Pentavalent 3 Vaccine among children aged 0–23 Months in Cameroon based on the Demographic and Health Survey 2018. PHMT. (2025) 16:35–45. doi: 10.2147/PHMT.S497787 PMC11830943 39963689

[24] DHS. (2026). The DHS Program - Cameroon: Standard DHS, 2018. Available online at: https://dhsprogram.com/methodology/survey/survey-display-511.cfm (Accessed June 27, 2026)

[25] WHO. (2026). Explorations of inequality: childhood immunization. Available online at: https://iris.who.int/items/6937a4bc-0062-4312-ba0d-b397e2e29937 (Accessed July 23, 2026)

[26] Hosmer DW Jr Lemeshow S Sturdivant RX. "Model-building strategies and methods for logistic regression". In: Applied Logistic Regression. Hoboken, New Jersey: John Wiley & Sons, Ltd (2013). p. 89–151.

[27] Mickey RM Greenland S. The impact of confounder selection criteria on effect estimation. Am J Epidemiol. (1989) 129:125–37. doi: 10.1093/oxfordjournals.aje.a115101, 2910056

[28] ICRC. (2021). Attacks on Health care. Available online at: https://www.icrc.org/en/document/health-care-providers-patients-suffer-thousands-attacks-health-care-services-past-5-years (Accessed August 4, 2026)

[29] International Committee of the Red Cross. (2026). Protection of Health care. Available online at: https://www.icrc.org/en/law-and-policy/protection-health-care (Accessed August 4, 2026)

[30] Haddison EC Ngono D Kouamen GT Kagina BM. Successful polio supplementary immunisation activities in a security compromised zone – experiences from the southwest region of Cameroon. Vaccine. (2018) 36:6961–7. doi: 10.1016/j.vaccine.2018.10.004, 30314909

[31] ReliefWeb. (2019). Cameroon: north-west and south-west crisis situation report no. 2 - as of 31 December 2018 - Cameroon. Available online at: https://reliefweb.int/report/cameroon/cameroon-north-west-and-south-west-crisis-situation-report-no-2-31-december-2018 (Accessed June 27, 2026)

[32] Vouking MZ Binde T Tadenfok CN Ekani JME Ekra D. Contribution of community health workers to surveillance of vaccine-preventable diseases in the Obala health district. Pan Afr Med J. (2017) 28:11537. doi: 10.11604/pamj.2017.28.207.11537 PMC5878844 29610645

[33] Bobo FT Asante A Woldie M Dawson A Hayen A. Child vaccination in sub-Saharan Africa: increasing coverage addresses inequalities. Vaccine. (2022) 40:141–50. doi: 10.1016/j.vaccine.2021.11.005, 34794824

[34] Birhanu F Yitbarek K. Wealth-based inequity in full child vaccination coverage: an experience from Mali, Bangladesh, and South Africa. A multilevel poison regression. PLoS One. (2023) 18:e0293522. doi: 10.1371/journal.pone.0293522 PMC10732374 38117824

[35] Forshaw J Gerver SM Gill M Cooper E Manikam L Ward H. The global effect of maternal education on complete childhood vaccination: a systematic review and meta-analysis. BMC Infect Dis. (2017) 17:801. doi: 10.1186/s12879-017-2890-y, 29281990 PMC5745980

[36] Miikkulainen A Abdirahman Mohamud I Aqazouz M Abdullahi Suleiman B Sheikh Mohamud O Ahmed Mohamed A . Antenatal care utilization and its associated factors in Somalia: a cross-sectional study. BMC Pregnancy Childbirth. (2023) 23:581. doi: 10.1186/s12884-023-05871-4, 37573367 PMC10422779

[37] Rossi R. Do maternal living arrangements influence the vaccination status of children age 12–23 months? A data analysis of demographic health surveys 2010–11 from Zimbabwe. PLoS One. (2015) 10:e0132357. doi: 10.1371/journal.pone.0132357 PMC4500504 26167939

[38] Ozigbu CE Olatosi B Li Z Hardin JW Hair NL. Correlates of zero-dose vaccination status among children aged 12–59 months in sub-Saharan Africa: a multilevel analysis of individual and contextual factors. Vaccines. (2022) 10:1052. doi: 10.3390/vaccines10071052 PMC9322920 35891216

[39] WHO. (2026) Immunization Agenda 2030. Available online at: https://www.who.int/teams/immunization-vaccines-and-biologicals/strategies/ia2030 (Accessed June 27, 2026)

[40] UNICEF Cameroon. (2026) Reaching every child: life-Saving Vaccines for conflict-Affected Communities in Cameroon. Available online at: https://www.unicef.org/cameroon/stories/reaching-every-child-life-saving-vaccines-conflict-affected-communities-cameroon-0 (Accessed July 23, 2026)

[41] UNICEF Cameroon. (2026). Annual Report 2024 _UNICEF Cameroon.Pdf. Available online at: https://www.unicef.org/cameroon/media/2691/file/ANNUAL%20REPORT%202024%20_UNICEF%20Cameroon.pdf.pdf?utm_source=chatgpt.com (Accessed July 23, 2026)

[42] UNICEF Cameroon. (2026). Cameroon-RTM-Case-Study. Available online at: https://www.unicef.org/wca/media/10481/file/Cameroon-RTM-Case-Study (Accessed July 23, 2026)

[43] WHO. (2026) Countries are already experiencing significant health system disruptions – WHO. Available online at: https://www.who.int/news/item/10-04-2025-countries-are-already-experiencing-significant-health-system-disruptions---who (Accessed July 23, 2026)

